# Rhizosphere hydrophobicity: A positive trait in the competition for water

**DOI:** 10.1371/journal.pone.0182188

**Published:** 2017-07-28

**Authors:** Thorsten Zeppenfeld, Niko Balkenhol, Kristóf Kóvacs, Andrea Carminati

**Affiliations:** 1 Department of Physical Geography, Institute of Geography, University of Goettingen, Goettingen, Germany; 2 Department of Wildlife Sciences, Faculty of Forestry and Forest Ecology, University of Goettingen, Goettingen, Germany; 3 Division of Soil Hydrology, Faculty of Agricultural Science, University of Goettingen, Goettingen, Germany; Estacion Experimental del Zaidin, SPAIN

## Abstract

The ability to acquire water from the soil is a major driver in interspecific plant competition and it depends on several root functional traits. One of these traits is the excretion of gel-like compounds (mucilage) that modify physical soil properties. Mucilage secreted by roots becomes hydrophobic upon drying, impedes the rewetting of the soil close to the root, the so called rhizosphere, and reduces water availability to plants. The function of rhizosphere hydrophobicity is not easily understandable when looking at a single plant, but it may constitute a competitive advantage at the ecosystem level. We hypothesize that by making the top soil hydrophobic, deep-rooted plants avoid competititon with shallow-rooted plants. To test this hypothesis we used an individual-based model to simulate water uptake and growth of two virtual plant species, one deep-rooted plant capable of making the soil hydrophobic and a shallow-rooted plant. We ran scenarios with different precipitation regimes ranging from dry to wet (350, 700, and 1400 mm total annual precipitation) and from high to low precipitation frequencies (1, 7, and 14 days). Plant species abundance and biomass were chosen as indicators for competitiveness of plant species. At constant precipitation frequency mucilage hydrophobicity lead to a benefit in biomass and abundance of the tap-rooted population. Under wet conditions this effect diminished and tap-rooted plants were less productive. Without this trait both species coexisted. The effect of root exudation trait remained constant under different precipitation frequencies. This study shows that mucilage secretion is a competitive trait for the acquisition of water. This advantage is achieved by the modification of the soil hydraulic properties and specifically by inducing water repellency in soil regions which are shared with other species.

## Introduction

Plant community composition is driven by competition among individuals for resources like light, nutrients, and water [1]. The acquisition of water resources by plants has risen interest of eco-hydrologists for example in context of improving food production [2] but also as an explanatory factor for emergence of self-organized vegetation patterns [3]. In the competition for water the root system provides the plant-infrastructure for water uptake and below-ground interactions. Relevant functional root traits include the morphological structure and configuration of the root system (e.g., root length, architecture). The root system architecture is characterized by spatial configuration, depth, branching, and length of roots [4]. It determines the amount, density, and distribution of active root-surfaces. This configuration shapes the hydraulic conductivity of the plant-soil system, which determines the ease of water-transport from the soil to the shoot.

Plants can manipulate the hydraulic conductivity by directed root growth or by actively manipulating the soil properties. The latter is, for instance, due to the exudation of photo-assimilates into the soil by plant roots. These root exudates typically consist of sugars, amino acids, organic acids and lipids [5]. They play an important role for nutrient uptake [6] and they actively shape soil-microbiological composition [5, 7]. In the context of plant water uptake, attention has been drawn on the hydraulic properties of root exudates-soil complexes [8]. Especially mucilage plays a significant role here. Mucilage is a gel-like root-secret which consists of high-molecular weighted compounds like polysaccharides and a small fraction of lipids. It is released at the root tips into the rhizosphere, a small volume of soil around the roots. Mucilage is able to store a high amount of water and, thus, maintains the rhizosphere wet and hydraulically conductive [9, 10]. On the other hand, mucilage becomes hydrophobic upon drying, causing a zone of water repellent soil in the vicinity of roots or rhizosphere hydrophobicity [11, 12]. Note that other origins of soil water repellency exist, such as decomposition of wax-rich plant litter or condensation of long-chained organic compounds after burning [13], but we focus in this study only on soil hydrophobicity caused by rhizodeposition and particulary mucilage secretion.

Although a wide range of plant species excrete mucilage [14], the resulting degree of hydrophobicity could vary. For instance, mucilage from maize (*Zea mays*) and lupins (*Lupinus albus*) is more hydrophobic than the one of barley (*Hordeum vulgare* or wheat (*Triticum aestivum*) [15, 16].

Beside this variability in mucilage water repellency, the ecological function of the rhizosphere hydrophobicity is unclear. At the single plant scale, in controlled laboratory experiments, rhizosphere hydrophobicity was found to limit water uptake after drying/wetting cycles for lupins [17, 18]. But why do plant roots invest energy in making the soil in their vicinity water repellent and thereby reducing their ability to take up water?

Our hypothesis is that rhizosphere hydrophobicity, in some circumstances, provides an advantage in the competition for water. We hypothesize that by making the topsoil hydrophobic, plants with deep roots avoid the competition with shallow-rooted plants that have no access to water stored in the subsoil.

**Fig 1:**
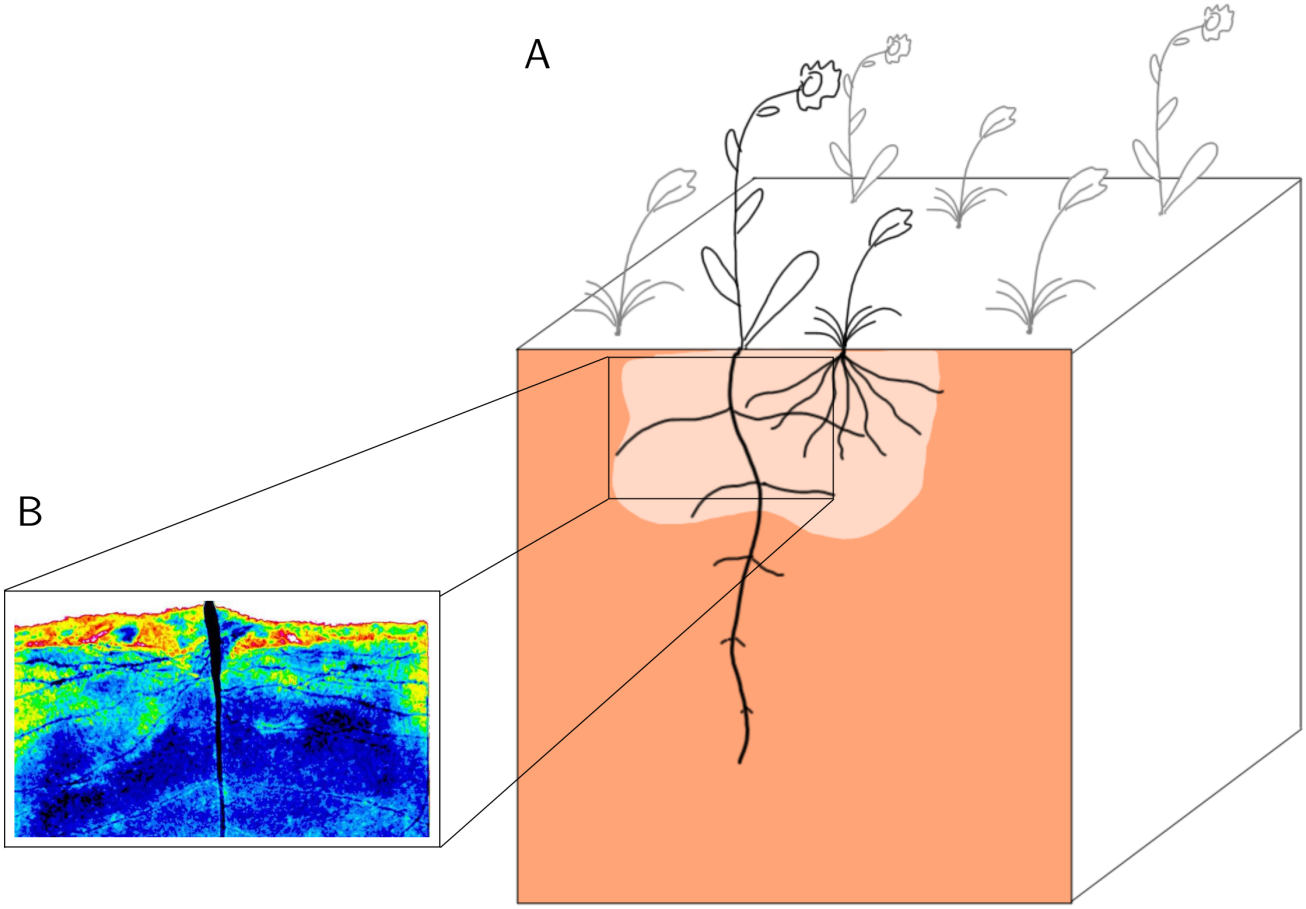
A conceptual visualization of the study system. A: The cross-section of the soil profile shows a hydrophobic zone in the topsoil (light brown). This zone is hypothesized to be induced by root mucilage, which becomes hydrophobic after drying. The inset B is taken from [20]. It shows the soil water distribution ranging from reddish–yellowish colors for dry soil to blue color tones for wet soil areas. Re-wetting after a drying period is inhibited in topsoil and only occurs in lower soil areas.

To fully understand such plant-soil and plant-plant interactions laboratory experiments with single plants (such as the experiments proving that the rhizosphere of selected plants turns hydrophobic upon drying) are not sufficient. Instead, analyses at the plant community level are required. Since large-scale field experiments are logistically challenging and face an increased complexity of the study system (e.g., genetic variability, above-ground interactions, competition for other resources such as nutrients, etc.), we took a different approach using an individual-based model (IBM). Specifically, we acquired information from laboratory experiments about root architecture and rhizosphere traits and included them in a spatially explicit individual-based population model. Individual-based simulation models gained importance as an experimental system to investigate complex patterns and processes in ecology [19]. Their particular strength lies in upscaling small-scale interactions, for example among and between abiotic and biotic entities like soil and plants, to the population scale. This is done by a ‘bottom-up’ approach in which an algorithm determines intra- and inter-specific behavior as well as resource dynamics in terms of usage, availability, and supply. We used this IBM to simulate growth and water uptake of two plant species with contrasting root and rhizosphere properties, one deep-rooted plant capable of exuding mucilage and making the topsoil hydrophobic and one shallow-rooted plant (Fig 1).

We hypothesized that by investing assimilates in mucilage a plant actively induces soil hydrophobicity, thus diminishing water availability for competitors. Despite its costs, root exudation should therefore be beneficial for the plant and its population.

## Materials and methods

We simulated growth of two different plant species with contrasting traits, a deep-rooted species exuding hydrophobic mucilage in the topsoil and a shallow-rooted species not exuding mucilage at all. By estimated the hydraulic conductivity of the root systems we parameterized water uptake and plant growth processes in the population model.

### Root systems

**Fig 2:**
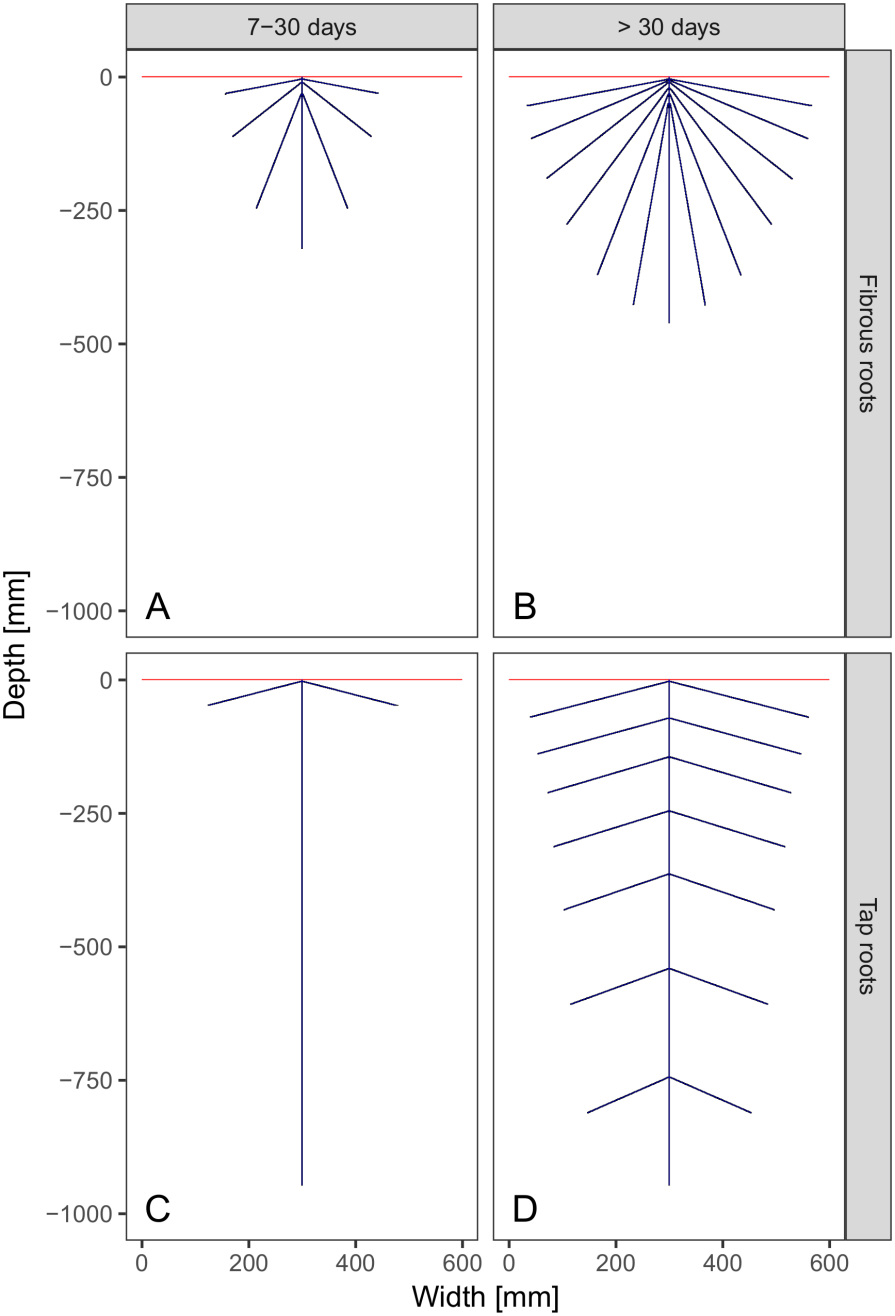
Root systems at different developmental stages. (A) Fibrous roots at intermediate stage show seven short adventitious root branches. (B) At final root growth stage, fibrous-rooted plants have 13 rootbranches. (C) Tap-roots after seven days show a principal vertical root reaching final depth already. Secondary root branches are restricted to one pair next to the surface. (D) The fully developed tap root system consists of the principal vertical root and seven pairs of lateral branches with a higher density in the topsoil. The root/soil system is capped by a impermeable barrier for water (red line) with one raster cell exit to the shoot/above ground system. For the first root growth stage (1–6 days) no water uptake was assumed and, hence, no sketch was drawn.

We described two different root systems by drawing their simplified architectures in a soil profile of 60 cm width and 100 cm depth (Fig 2). The first, fibrous, root system is characterized by 13 adventitious root branches without hierarchical order which originate at the shoot axis and spread into the topsoil only. Fibrous roots are commonly found in monocotyledon plant species like maize [21]. The second root system is a tap root system with one deep, vertical root and seven pairs of secondary lateral root branches. Tap root systems are common in dicotyledon plant species [21].

Each root system was described at three different growth stages: 1) Initial seed germination stage: in the first week we assumed that the seeds germinate and establish a first principal root. At this stage we assumed that there are no physiognomical differences between tap and fibrous root systems. Further, we assumed that transpiration is negligible. 2) After the first week an intermediate stage follows wherein fibrous roots already show their final architecture, but branches do not penetrate deeper soil areas (Fig 2A). Tap roots in this stage establish their principal vertical root and first laterals (Fig 2C). The first and intermediate stages were assumed to last one month. 3) The final root growth stage depicts the fully developed root system (Fig 2B–2D). It was supposed to be reached after one month of growth and lasts to the death of the individual.

We assumed the same total root length for drawing fibrous and tap systems at each root growth stage (stage 2: 129.7 cm, stage 3: 391.9 cm) to guarantee comparability between both.

### Hydraulic resistance

Water transport from the soil matrix via roots, plants xylem, and leaves into the atmosphere is commonly described by analogy with electric circuit [22, 23]. Therein each transition (e.g., soil–root xylem) along the water pathway is treated like an electrical (*sensu* hydraulic) resistor with a specific resistivity. Accordingly, our soil-plant system would be a circuit consisting of resistors for soil with a certain water saturation, root cortex, and root stele. The major benefit of applying this analogy is, that the total effective hydraulic resistance of the soil-plant system can be quantified by applying Ohm’s and Kirchhoff’s circuit laws. This total effective hydraulic resistance is proportional to the work a plant has to afford to move water from soil to the shoot. The hydraulic resistance depends on root system architecture, the resistance of cortex and stele elements, and on soil water conditions.

In order to quantify the total effective hydraulic resistance of the two root systems we first had to assign hydraulic resistivities. The setup consisted of a rasterized version of the root system sketches (raster cell dimension: 1x1 mm) as shown in Fig 2. The root branches were reduced to a width of one raster cell (skeletonization algorithm) which we asssigned to the root stele, the central cylinder, where water flows axially to the shoot. The entire stele was enclosed by the cortex, a layer with a width of one raster cell. In order to direct water flux to the aboveground shoot of the plant the soil surface was capped by a impermeable layer (resistivity = ∞). Only stele penetrated this layer to connect to the aboveground plant shoot. We assigned hydraulic resistivity values to each raster cell according to current physiological knowledge of root development. For the cortex we imposed a linear resistivity gradient declining with soil depth to account for higher hydraulic conductivity of younger parts of the root system. This is based on the assumptiion that older roots are less effective in water-uptake because their cortex becomes thicker with secondary growth. In contrast, resistivities of stele cells increased with soil depth to guarantee an upward flow of water to the shoot.

For calculating the total effective hydraulic resistance of the root system we used the software *circuitscape* [24] (for details see S1 Text). The resulting total hydraulic resistance at a given soil water saturation is proportional to the difference in water potential between soil and root xylem. It can be seen as a measure of the physical work the above-ground part of the plant has to do to extract soil-water. We fitted a water-uptake function for each root system at each growth stage as a function of derived resistance values at different soil water conditions. We did not calculate hydraulic resistances for the initial germination root growth stage (first week), as we did not assume any relevant transpiration in this phase.

The objective of these calculations was to quantify how capable the two root systems are in taking up water when the soil dries. Soil drying was simulated by increasing the resistivity of the soil matrix. These resistivity values at a given soil water saturation (Θ) were parameterized using the Van Genuchten model [25]:

$$\begin{array}{c} K_{r}\left(\Theta \right)=\Theta^{{}^{1}{/}_{2}}{\left[1-{\left(1-\Theta^{m}\right)}^{m}\right]}^{2} \end{array}$$ (1)

with $m=1-\frac{1}{n}$ and *n* is set to 2. The hydraulic resitivity of one soil raster cell is $R=\frac{1}{K_{r}}$. A detailed description is given in Supplement S1 Text.

Under conditions with no hydrophobicity, resistivity decreases with soil depth, because deeper soils are typically wetter. In case of hydrophobicity, resistivity values of the topsoil (0–50 cm) are high and shift abruptly to low values in the subsoil.

#### Water uptake & vitality

Water uptake by plants is inversely related to the total hydraulic resistance. Therefore, we defined a species-specific state variable *Vitality*. It is the normalized inverse of the total hydraulic resistance at a given soil water saturation (and root system architecture). Its values range from 1 to 0: when *Vitality* = 1 the total resistance is low and the plant’s water uptake is maximum. In case of absence of hydrophobicity we parameterized *Vitality* as a function of total soil water saturation Θ_*total*_ averaged over the entire soil profile of 1 m. When hydrophobicity was active, *Vitality* is a function of top- and subsoil water saturation (Θ_*top*_, Θ_*sub*_), as explained below. Parameterization was done by using minpack.LM in R [26].

### Individual-based model

The following description of the model is an excerpt of the ODD protocol (Overview, design concept and details) [27, 28]. The complete protocol is given in supporting information S2 Text. The model was written in NetLogo (Version 6.0) [29]. The corresponding source code is provided in S1 File.

#### Purpose

We hypothesized that a plant profits by investing in root exudates and manipulating herewith water availability. In an individual-based model we upscale these small-scale functional trait effects to test competition between two species of varying root traits at population level.

#### Entities

The model comprises individuals of two plant species with different root architectures: tap roots and fibrous roots. They are located on patches which are considered to be soil entities of homogeneous physiochemical character (i.e., composition, texture, depth etc.).

#### State variables

To each soil patch a water saturation (Θ = 0–1) is attributed, defined as the share of water-filled pore volume on total soil pore volume. In presence of tap-roots with an active trait of root exudation, Θ is split into Θ_*top*_ for the topsoil (0–50 cm) and Θ_*sub*_ for the subsoil (50–100 cm) and treated separately. Variable plant attributes are biomass, as a result of a growth process, and vitality, a species-specific function of soil water saturation. Biomass has an impact on individual’s transpiration, its growth rate, and its reproduction. The sum of biomass per soil patch influences the success of a reproduction event and, in case of tap-roots, the amount of exudates deposited in the soil. Vitality has an impact on mortality, growth, and reproduction of an plant individual. Tap-rooted plants could switch to a hydrophobicity modus, wherein they expend a certain amount of assimilates to exude mucilage and turn the topsoil hydrophobic.

#### Scales

Each patch is considered to be a 1x1x1m volume of homogenously textured soil. The whole simulation landscape extends to 101x101 patches (∼1 hectare). One simulation time step comprises a period of one day.

#### Process overview and scheduling

**Fig 3:**
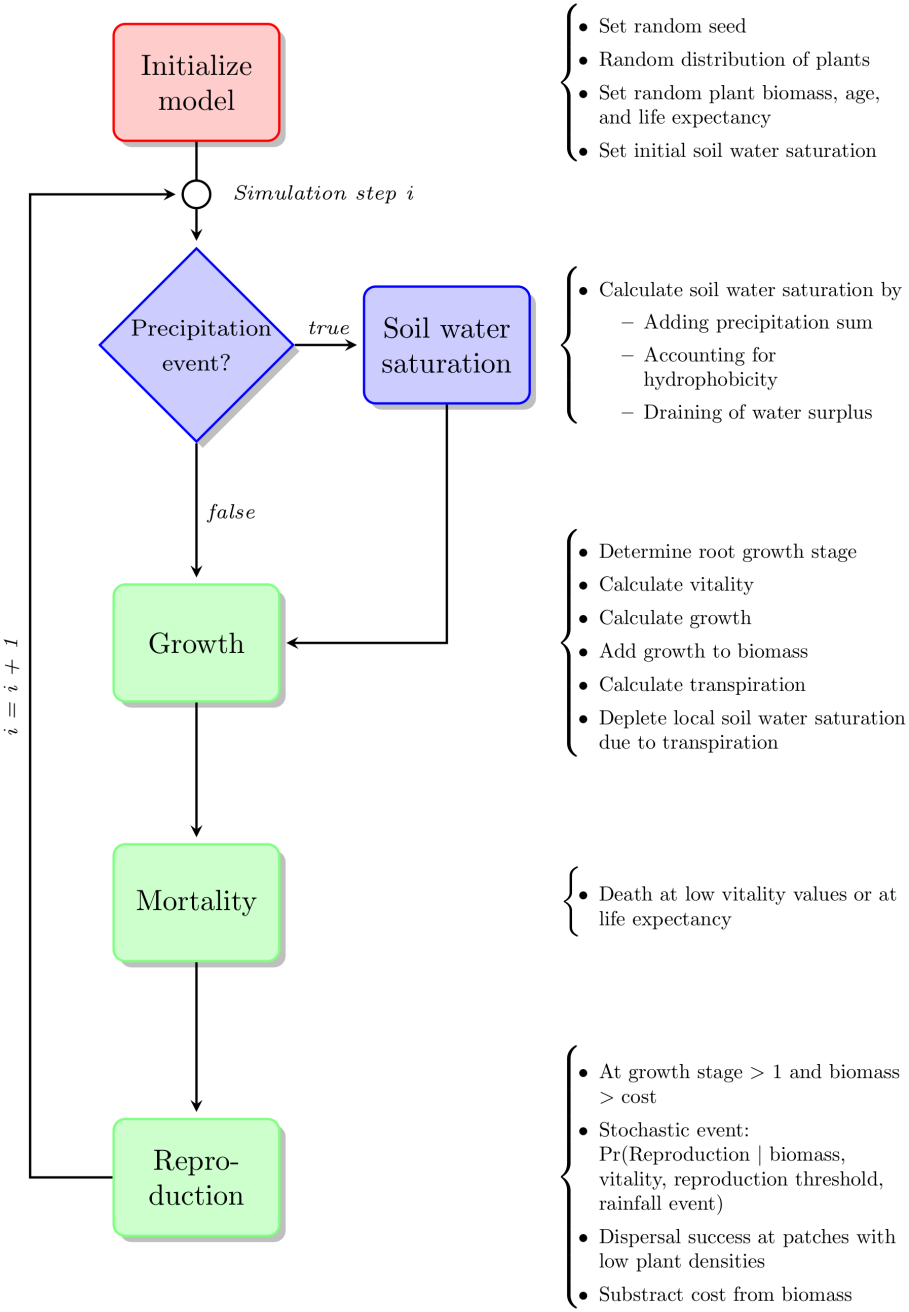
Basic flowchart of the model. A global process (Initialization) is colored in red, (soil-)water related processes are filled in blue, and plant processes are given in green.

After initialization the model successively runs a series of soil and plant related processes (Fig 3). At the beginning of each simulation step, a precipitation event provides the system with a certain amount of water. For each soil patch a water saturation value is calculated with respect to a potential hydrophobic regime in case a tap-rooted plant is present. According to this local soil water saturation, for every plant individual the *Vitality* value is calculated. *Vitality* is regulatory for succeeding processes of growth, mortality, and, along with others, for reproduction probability.

#### Soil water saturation

At the beginning of each simulation step *i* the water saturation Θ_*i*_ of each soil patch is calculated. In the absence of root exudates (either by deactivated modus or by absence of tap-rooted plants) this is straight-forward by adding the precipitation sum *P*_*i*_ to the previous water saturation Θ_*i*−1_:

$$\begin{array}{c} \Theta_{i}=\Theta_{i-1}+\frac{P_{i}}{\phi \cdot z} \end{array}$$ (2)

where *ϕ* = 0.5 is soil porosity and *z* is the depth of the soil profile. When Θ_*i*_ exceeds 1 it is set to 1 and the additional water is assumed to precolate deeper then 1 m.

If a tap-rooted plant is present on the soil patch and the exudation trait is activated, soil water saturation of this particular patch is calculated differently. The water saturation is treated separately for topsoil and subsoil. The distribution of precipitation water *P_*i*_* between top- and subsoil depends on a factor *ω*_*i*_.

$$\begin{array}{c} \Theta_{top,i}=\Theta_{top,i-1}+\frac{P_{i}}{\phi \cdot z_{top}}\cdot (\frac{1-\omega_{i}}{2-\omega_{i}}) \end{array}$$ (3)

$$\begin{array}{c} \Theta_{sub,i}=\Theta_{sub,i-1}+\frac{P_{i}}{\phi \cdot z_{sub}}\cdot \frac{1}{2-\omega_{i}} \end{array}$$ (4)

The distribution factor *ω*_*i*_ ranges from 0 to 1 and describes the share of the top- and subsoil on incoming precipitation water. The more mucilage is exuded from tap-roots, the more water is repelled from this region and drained to the subsoil (*ω*_*i*_ → 1). If *ω* = 0 (no hydrophobicity), precipitation water is equally shared in top- and subsoil. The amount of mucilage per soil patch is proportional to the total root biomass of tap-rooted plants in the soil volume.

We described the distributional factor as follows:

$$\begin{array}{c} \omega_{i}=\frac{Biomass_{roots,i}\cdot \pi \cdot r_{rhizo}^{2}}{2\pi r_{root}^{2}\cdot \rho_{root}\cdot V_{topsoil}} \end{array}$$ (5)

with *Biomass*_*roots*,*i*_ being the below-ground share (0.5) of total tap-rooted plant biomass. *r*_*rhizo*_ = 0.4 cm is the radius of rhizosphere, *r*_*root*_ = 0.02 cm is the radius of a root branch, *ρ*_*root*_ = 0.01 *g*/*cm*^3^ is the density of a root branch, and *V*_*topsoil*_ = 50x100x100 cm^3^ is the total volume of the topsoil.

#### Initialization

As initial setup either a tap- or a fibrous-rooted plant is distributed on each soil patch. This results in an average abundance of 5100 individuals per species. They are attributed with a random age (1–400 d), life expectancy (*N(μ* = *600 d*, *σ* = *100 d)*), and biomass ($\frac{Age}{Expectation}\cdot max\_biom$). To each soil patch an initial water saturation Θ is given, which is drawn from a normal distribution with mean 0.5 and standard deviation 0.2.

#### Growth

Plant growth is supposed to be proportional to total biomass of the individual and its *Vitality*. We assumed that the growth rate inclines when the plant reaches a maximum biomass.

Plant’s biomass increment was calculted by a logistic growth function:

$$\begin{array}{c} {Biomass}_{i}={Biomass}_{i-1}+\frac{{Vitality}_{i}}{50}\times {Biomass}_{i-1}\cdot \left(1-\frac{{Biomass}_{i-1}}{max\_biom}\right) \end{array}$$ (6)

As taprooted plants have to invest in mucilage exudation, 20% (11%–27% [30]) of their photoassimilates (determined by the growth function) are not contributing to biomass increment.

According to the coefficient of transpiration ($800\;\frac{gH_{2}O}{gCO_{2}}$) the amount of transpiration water is calculated by the growth. In case of tap-rooted plants, transpiration water is taken in equal shares from top- and subsoil. Fibrous-rooted plants, which have shallower roots, do only take up water from the topsoil. This means that in presence of tap-rooted plants Θ_*top*_ and Θ_*sub*_ is depleted through transpiration, while fibrous-rooted plants only deplete Θ_*top*_.

#### Mortality

There are two reasons for death of an individual: either its vitality drops below the mortality threshold of 0.2 or its age reaches life expectancy.

#### Reproduction & Dispersal

Reproduction is implemented as a stochastic event. In each simulation step for each individual with *age* > 30*d* a random number from 0 to *rep_threshold* (1000) is drawn. If this number is smaller than the product of *Biomass*^3^ and *Vitality*, a dispersal event is commenced. Dispersal is simulated by a simplified Gaussian kernel, where a random angle is chosen and the dispersal distance is a realization of a Gaussian normal distribution with *μ* = 0 and *σ* = *seed*_*dist*. If the patch in the dispersal distance shows a total *biomass* < 10 a new seedling is initialized with *biomass* = 1, which is also the cost for the parent plant.

### Scenarios

We tested the performance of root-specific traits in the competition of two plants for below-ground water resources. For this we manipulated amount and timing of water input (resp. precipitation) in the system. We ran three scenarios (*dry*, *moderate*, and *wet*) with an annual precipitation sum of 350 mm, 700 mm, and 1400 mm. Furthermore, we simulated three different precipitation frequencies, a continuous rainfall, a scenario with a moderate drying period (7 d), and one with an extended drying period (14 d).

### Simulation runs & Analysis

Each run lasted 3650 steps, which matches a simulated time period of 10 years. As the model includes a number of stochastic processes, we ran 100 repetitions with different random seeds.

We explored emergence of spatial patterns due to intra- and interspecific interaction. We tested if point patterns determined by the location of the plant individuals at successive simulation steps deviate significantly from the null model of complete spatial randomness (CSR). In particular we calculated a pair correlation function for tap- and fibrous-rooted individuals separately as well as a cross pair correlation function for both species [31]. The pair correlation function *g(r)* calculates the propability of occurrence for a pair of points separated by distance *r* normalized by the probability of Poisson point process under CSR [32]. Its values indicate complete randomness when *g*(*r*) = 1, attraction or aggregation when *g*(*r*) > 1, and inhibition or scarcity when *g*(*r*) < 1 at a given distance *r*. These functions were compared to 199fold Monte Carlo simulations of point patterns following CSR. These simulations form a confidence envelope. If the observed pair correlation function falls outside the envelope at a given distance *r* it indicates a significant non-random pattern. Statistical analyses (and graphical display) were conducted in R [26] using package spatstat [32].

## Results

### Hydraulic resistance

**Fig 4:**
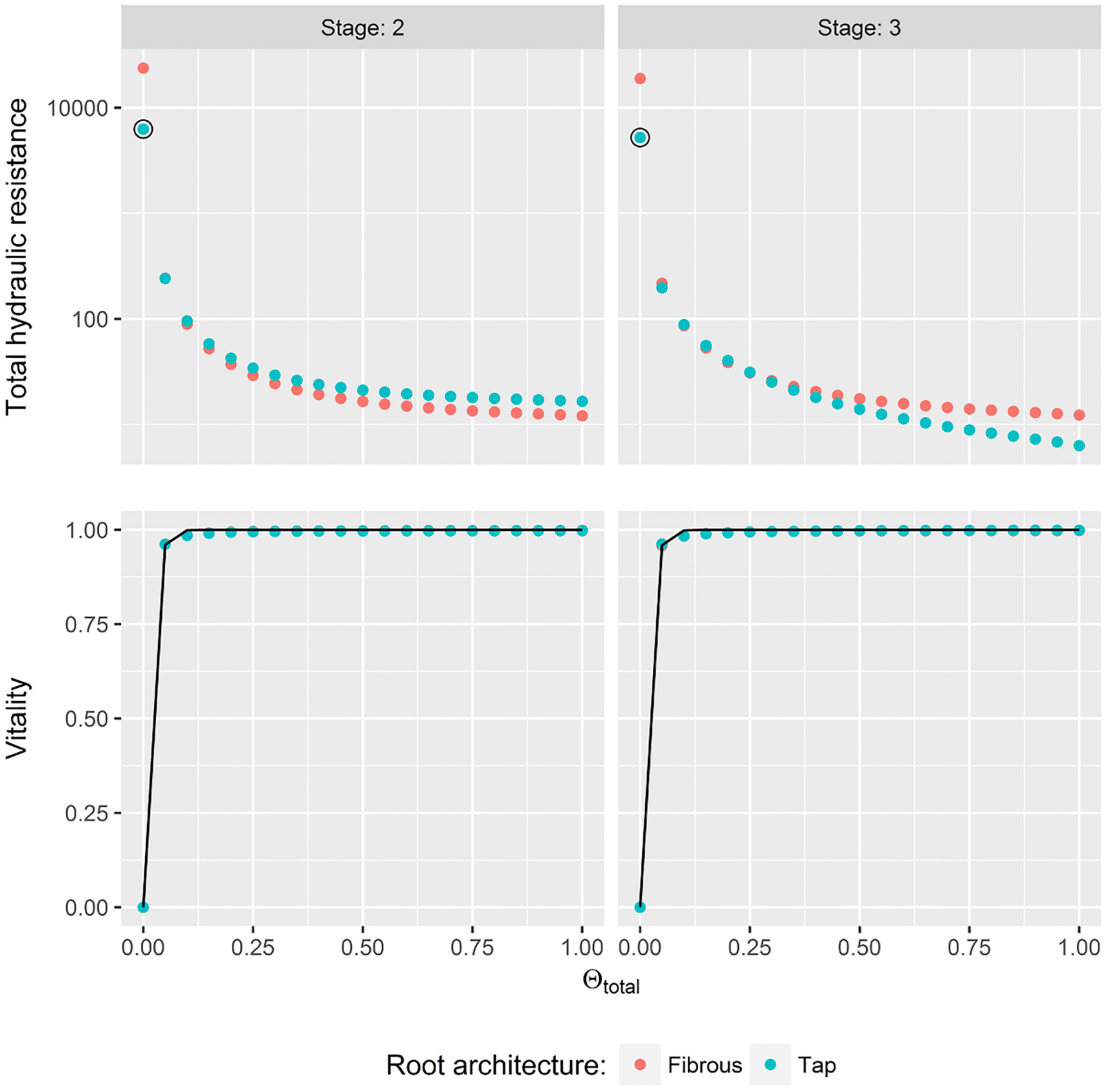
Quantification of water-uptake by plants according to hydraulic resistances at different soil water saturation levels. In the top row total hydraulic resistances of plants at different root system developmental stage (columns) in relation to total soil water saturation Θ_*total*_ (without hydrophobicity) are displayed. Note the logarithmic y-axis. In the bottom row normalized inverse of the resistance values are displayed as they were used to fit a vitality function (line). Reference value for normalization was the second highest resistance value at Θ_*total*_ = 0 (black circle).

The total hydraulic resistance of the two root systems did not differ substantially at both development stages (Fig 4). Only for Θ_*total*_ < 0.1 hydraulic resistance was high and *Vitality* dropped to zero. With higher soil water saturation both plant types showed maximum *Vitality* values.

As a consequence we fitted *Vitality* values by a function with asymptotic behavior:

$$\begin{array}{c} {Vitality}_{i}\left(\Theta_{total}\right)=\frac{1-e^{-k\cdot \Theta_{total,i}}}{1-e^{-k}} \end{array}$$ (7)

Best fitting parameters were *k* = 98.79 for root stage two and *k* = 63.88 for root system at stage three.

**Fig 5:**
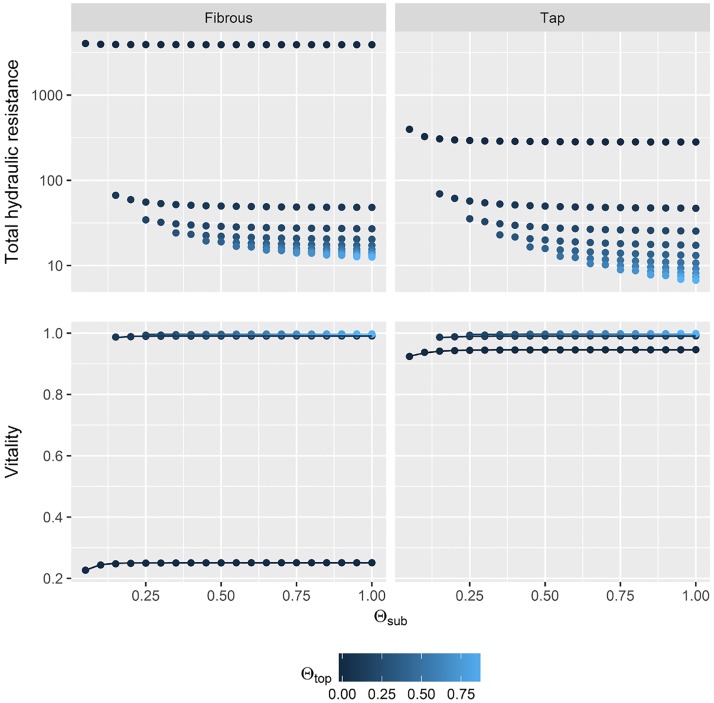
Quantification of water-uptake by plants at hydrophobic soils according to hydraulic resistances at different soil water saturation levels. In the top row total hydraulic resistances of plants with different root system architectures (columns, development stage 3) in relation to subsoil water saturation Θ_*sub*_ and to different strengths of hydrophobicity affecting the water saturation of the topsoil (Θ_*top*_). Note the logarithmic y-axis. In the bottom row normalized inverse of the resistance values are displayed as they were used to fit a vitality function (line).

When hydrophobicity is present in the topsoil, plant root systems showed different total hydraulic resistances (Fig 5). Especially when water content of the topsoil was dry (Θ_*top*_ = 0) fibrous-rooted plants faced high hydraulic resistances and water-uptake was impeded (*Vitality* = 0.25).

The corresponding species-specific *Vitality* function at given water contents in topsoil and subsoil (Θ_*top*_, Θ_*sub*_) were:

$$\begin{array}{c} {Vitality}_{i}\left(\Theta_{top},\Theta_{sub}\right)=1-e^{a\cdot \Theta_{top,i}+b}\cdot \frac{1}{1+e^{c\cdot \Theta_{sub,i}+d}} \end{array}$$ (8)

Here, four parameters had to be fitted (Table 1).

**Table 1:** Best estimates for parameters of Eq 8.

Root type	Stage	a	b	c	d
Fibrous	2	-52.70	-0.00	-3.49	-5.21
Tap	2	-33.17	-2.35	-118.71	0.32
Fibrous	3	-48.64	-0.29	-30.08	-0.74
Tap	3	-20.00	-2.96	-15.16	-3.03

### Simulation

**Fig 6:**
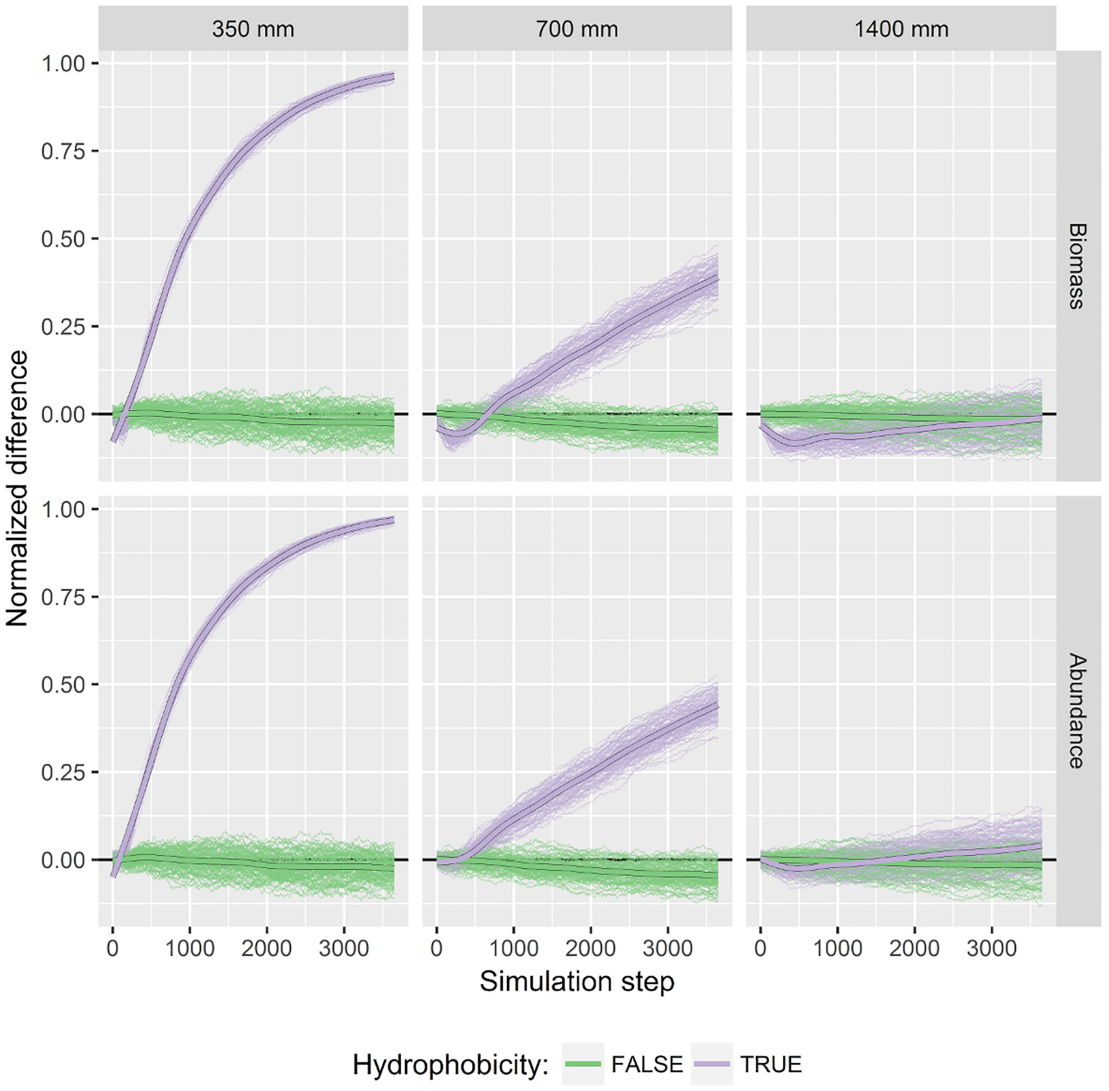
Impact of precipitation on plant populations with and without hydrophobicity in the soil. Columns show different precipitation sums. Rows show population indices total biomass (top panel) and total abundance (bottom panel). Precipitation frequency is seven days. Values are differences between tap-rooted to fibrous rooted plants. Differences are normalized by the total sum. Positive values are in favor of tap-rooted plants. Thin lines are results of 100 simulation runs with either hydrophobicity trait activated (violet) or deactivated (green). Thick lines are smoothed (spline) averages.

When the root trait of mucilage secretion was deactivated both plant species coexisted with none being dominant (Fig 6). This was observed throughout all simulation scenarios.

However, as soon as exudation started, the tap-rooted plant population benefited in abundance and–despite the investments of assimilates–biomass and its dominance increased with time. This positive effect for tap-rooted plants was most pronounced for the driest precipitation scenario (highest difference with 350 mm annual precipitation sum). In the wet scenario with 1400 mm annual precipitation sum the beneficial effect of root exudation did not appear.

Precipitation frequency, hence duration of dry periods, had impact on the intensity or quality of the trait effect (S1 Fig). At low precipitation frequencies (7 and 14 days) the hydrophobic trait had a strong effect on the relative difference between species. In case of high precipitation frequencies (each day) the difference between having a hydrophobic trait or not was smaller. This was the case for both scenarios with low and medium precipitation sums (350 and 700 mm).

**Fig 7:**
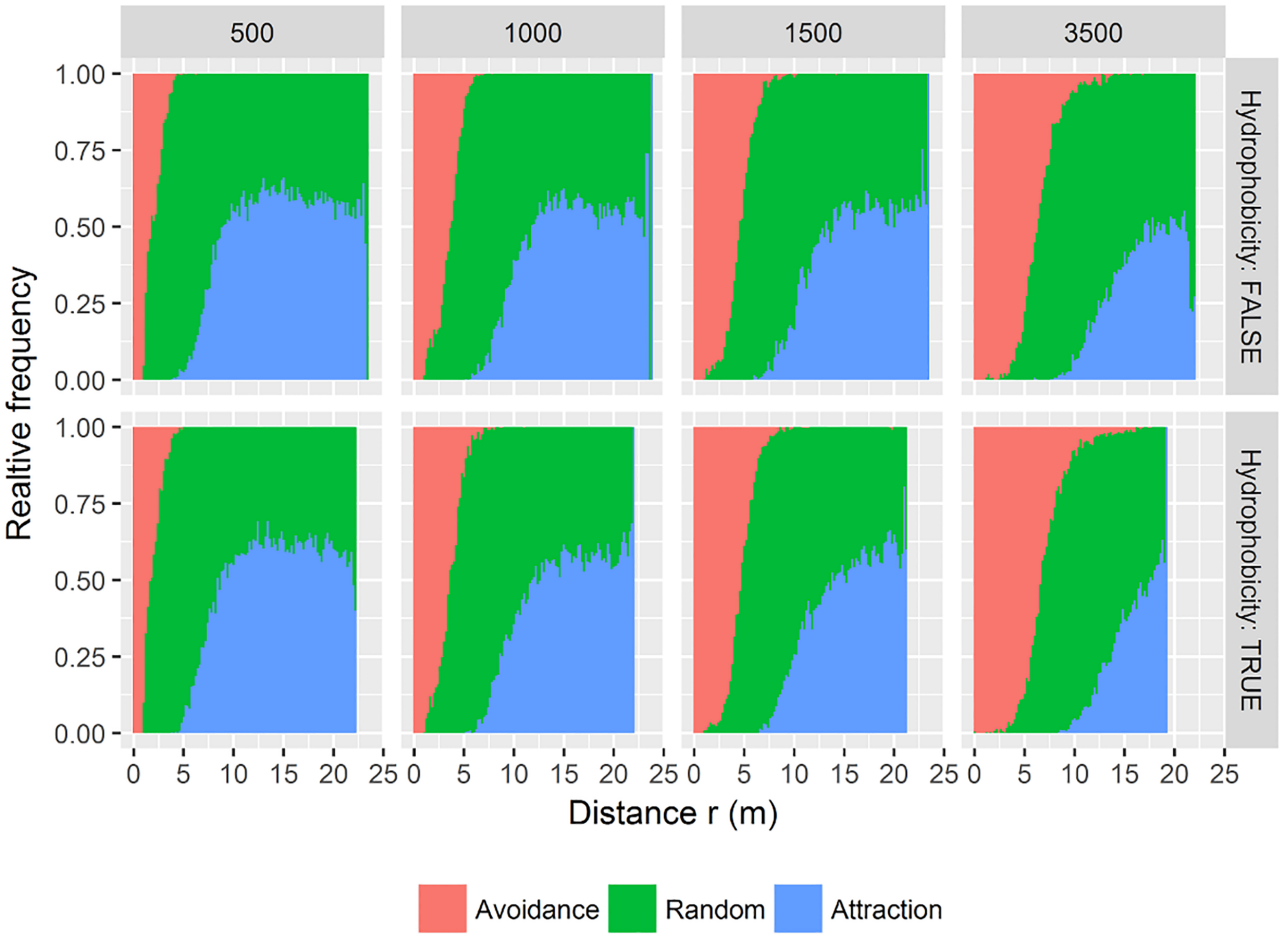
Scale-dependent spatial interactions between taproots and fibroots. Each panel shows the relative frequency of the outcomes of 199fold simulations of the pair-correlation functions. In each simulation run, the actual point pattern is tested against the null model of complete spatial randomness (green). Red colors indicate significant avoidance and blue colors show attraction compared to the null model. Statistics were run for four simulation steps: 500, 1000, 1500, and 3500 (columns) and two scenarios (without and with hydrophobicity (rows)). Precipitation frequency was seven days with an annual sum of 700mm.

Spatial organisation of plant individuals did not differ between simulations with and without rhizosphere hydrophobicity being active (Fig 7). In both scenarios spatial avoidance between species occurred only at small distances (< 5 m), which is still in the range of seed dispersal.

## Discussion

Our simulation experiment showed that investment of assimilates into root exudates (mucilage) can provide a benefit in interspecific competitiveness for water. An unfavorable condition like hydrophobicity in the topsoil–resulting from dried mucilage–turned out to be a competitive advantage for deep, tap-rooted plants over shallower fibrous-rooted plants. With the help of an individual-based model we were able to transfer this small-scale root trait effect to a large-scale population level.

### Modelling approach

Our modeling framework required a series of simplifications. In the water budget, for instance, we neglected important processes like evaporation and run-off. We also simplified the dynamics of vertical water distribution in the soil profile. We decided to keep the model as simple as possible to draw causal conclusions, but also as complex as necessary to include all relevant mechanisms. This allowed us to focus on pure root trait-related effects on the plant populations. For instance, the effects of root architecture and decreased water saturation in the topsoil induced by water repellency were explicitely included in the model, as they were the microscopic ingredients that emerged on the plant population dynamics on the plant scale. Overall, the setup was chosen to push hydrophobicity to an extreme. For example, we assumed a rather large area in the first 50 cm of the soil to be effected by root exudates. In fact, mucilage is released by root tips at all depths. However, rhizosphere hydrophobicity only in the topsoil was assumed due to commonly highest density of root tips (and mucilage concentration) in the topsoil. Further, mucilage turns hydrophobic upon drying, which is expected to occur more often in the upper 50 cm rather than in the wetter subsoil. Additionally, mucilage hydrophobicity was observed to be more marked around old root segments [20], which suggests that mucilage hydrophobicity increases over time and therefore it is likely to be stronger in the topsoil, where roots are older. Another factor to support a strong hydrophobicity effect was that we imposed the water repellent zone to persists as long as tap-rooted plants are present at the soil patch. Actually, water repellency diminishes as soon as mucilage re-hydrates (e.g. after 60 hours after re-wetting under laboratory conditions according to Carminati et al. 2010 [11]).

Finally, the assumption that the simulated shallow-rooted species does not exude hydrophobic mucilage is another simplification that might not reflect all possible scenarios and species interactions. Apart from these simplifications, the modelling approach is inevitable to gain insights into the functional role of rhizosphere hydrophobicity in plant competition.

### Rhizosphere hydrophobicity: A functional side-effect of root exudates?

Rhizosphere hydrophobicity adds a biophysical perspective to manifold ecological views on trait-based competition for resources. It is beneficial for deep-rooted plants for outcompeting shallow-rooted plants in the extraction of water from the top soil. Hydrophobicity is caused by lipids excretes into the soil [33]. Such lipids have additional functions: they allow an easier soil penetration and they decrease the surface tension of the soil solution, which enhances the drainage of large pores (important in case of waterlogging conditions) and facilitates the formation of films around soil particles and root hairs. Rhizosphere hydrophobicity–as a result of lipid concentration in dehydrated mucilage–might be a side-effect of the evoluted mutualistic trait.

Our results support the beneficial effect of rhizosphere hydrophobicity on population level when plant species with contrasting root architectures are present. However, we assumed that only one species showed sercretion of hydraulic active mucilage, while fibrous-rooted species also exude mucilage. Probably, for such species rhizosphere hydrophobicity does not bring apparent advantages. In addition, there are systems where only one root system architecture is dominant, for example grass dominated ecosystems or monoculture agricultural fields. Here rhizosphere hydrophobicity would mean self-inhibition with no competitional benefit on population level. For example in New Zealand pastures with water-repellent soils showed about half of the productivity in comparison to the control [34]. In practice, amelioration efforts are undertaken to reverse water-repellency in arable soils [35]. In summary, in such single-species ecosystems rhizosphere hydrophobicity reduces soil water resources and becomes a self-inhibition system. Interestingly self-inhibition due to unavailable water resources in a dryland-grass ecosystem has been proposed as a mechanism to describe emergence of self-organized vegetation patterns like ring or banded structures [36].

### Rhizosphere hydrophobicity versus hydraulic lift

Our modelling study showed that rhizosphere hydrophobicity results in competitive advantage of deep-rooted plants against shallow-rooted plants. However, there are also positive interactions between plant species with contrasting root architectural traits. One of such interactive mechanisms is known as hydraulic lift. Hydraulic lift refers to the transport water from the deep and wet soil regions to upper and drier soil layers through the root systems [37]. Via hydraulic lift, deep-rooted plants might therefore increase water availability to shallow rooted plants that have access only to soil water in the top soil. In contrast to secretion of mucilage, hydraulic lift is a passive process. It is the result of low transpiration (e.g., at night) and a higher water potential in roots than in the topsoil [37]. The two mechanisms of rhizosphere hydrophobicity and hydraulic lift have therefore opposite effects on the interactions between plants: one increasing competition and the other improving coexistence. Which process is more relevant? Probably it depends on the soil properties.

In coarse-textured soils, which have a small specific surface area, mucilage has a stronger effect on water repellency [38]. Additionally, rhizosphere hydrophobicity will decrease the hydraulic connectivity between roots and soil, decreasing the efflux of water from the roots at night and counteracting hydraulic lift. Therefore, it is likely that in coarse-textured soils the competitive mechanism of rhizosphere hydrophobicity becomes dominant.

In contrast for finer-textured soils mucilage coating is expected to be less effective and here hydrophobicity plays a minor role. Instead, hydraulic redistribution of soil water by roots is more likely to shape plant interaction in fine-textured soils [37].

### Species composition and pattern emergence

In our simulation rhizosphere hydrophobicity did not lead to spatial self-organization and, hence, to emergence of vegetation patterns. However, small-scale interactions could lead to emergence of vegetation patterns at larger scales. One unifying mechanism to explain this spatial self-organisation is the scale-dependent feedback [39]. It is effective when positive and negative feedback mechanisms (like facilitation and competition) occur on different spatial scales. Prominent examples are striped, labyrinth-like vegetation patterns in drylands called ‘tiger bushs’ [40]. Here, pattern emerge as a result of a small-scale positive feedback of plant biomass and water availability (higher infiltration, lower evaporation). In contrast distant regions of bare soil do not re-wet during a precipitation event due to hydrophobic biological crusts. In our simulation model only a negative feedback mechanism was present: in a small range around tap-rooted plants water became scarce due to rhizosphere hydrophobicity and this effect enforced with more tap-rooted plants being present. Rhizosphere hydrophobicity did not result in spatial patterning, probably because it was counteracted by dispersal.

### Small scale rhizosphere interactions shaping plant communities on population level

We proposed a rather indirect influence of exudates as a functional trait for shaping plant-plant interaction. In this study we focused on an example of rhizosphere interactions, the excretion of mucilage and the consequent water repellency of the rhizosphere upon soil drying. However root exudation has many other complex functions. Root exudates could directly affect competitors, either positively by stimulating root growth, attracting symbionts, or repelling parasites or negatively by using phytotoxines to inhibit growth of con-specific (autotoxcity) or inter-specific plants (allelopathy) [41]. Previous work focused on root exudates shaping biotic interactions between plant and microbiota (including mycorrhizal fungi) [4]. This mutual interaction affects for example the accessibility to nutrients, plant health or allelopathic plant-plant interactions [42, 43]. Since very early in terrestrial plant evolution, root exudates played a crucial role as an attractor for symbiontic mycorrhiza [44]. Physical effects of root exudates include root growth (easier penetration of bulk soil), soil structure (stabilization of particles, formation of rhizosheaths), and increased hydraulic conductivity during drying [10, 45].

All these complex and interacting processes take place in a thin layer of soil surrounding the roots, the rhizosphere, where most of microbial activity takes place, and which is a crucial biological and hydrological hot spot [46]. To predict how such fundamental small-scale processes impact plants at a larger scale, these processes have to be included in larger scale models where interactions between different plant species are considered. Here, we focused on an example of such interactions, rhizosphere hydrophobicity, which at the single plant level is expected to negatively impact water resources, but in the context of different plant species competing for water becomes a positive trait.

Including in such a conceptual model additional biogeochemical rhizosphere processes as well as above ground interactions would allow a better understanding of the role of microscopic interactions in the rhizosphere at the ecosystem level and compare single- with multi-species ecosystems.

## Supporting information

S1 Text Documentation of hydraulic conductivity determination. (PDF)

S2 Text ODD-Protocol of the model. (PDF)

S1 File NetLogo file of the model. (NLOGO)

S1 Fig Impact of precipitation intervals on biomass plant populations with and without soil hydrophobicity. Columns show different precipitation frequencies. Rows show population indices total biomass (top panel) and total abundance (bottom panel). Precipitation sum is 350 mm (A), 700 mm (B), and 1400 mm (C). Values are differences between tap-rooted to fibrous rooted plants. Differences are normalized by the total sum. Positive values are in favor of tap-rooted plants. Thin lines are results of 100 simulation runs with either hydrophobicity trait activated (violet) or deactivated (green). Thick lines are smoothed (spline) averages.
(PDF)
