# Supplementary material for: Rhizosphere hydrophobicity: A positive trait in the competition for water
Source: PLoS One. 2017 Jul 28;12(7):e0182188. doi: 10.1371/journal.pone.0182188 (PMC5533451; doi:10.1371/journal.pone.0182188)
Supplement: S1 Text — (PDF) [file pone.0182188.s001.pdf]

S1 Text: Hydraulic Resistance

Thorsten Zeppenfeld

Supporting information *S1 Text* of the publication: Zeppenfeld et al. 2017 *Rhizosphere Hydrophobicity: a Positive Trait in the Competition for Water*

Objective

In the simulation model of Zeppenfeld et al. (2017) a function relates *vitality* of a plant indivdual to the actual water saturation in the soil. *Vitality* describes the water-uptake by a plant and it is an important input value for other plant-related processes like growth, reproduction, or mortality.
The *vitality* function is dependent on hydraulic performance of the root system architecture at different soil-water saturations. We quantified hydraulic performance of the two different root architectures by calculating their total effective hydraulic resistance. This was done with the software *Circuitscape* (V. Shah and McRae 2008), a tool that originates in ecological landscape analysis and assesses the functional connectivity between populations and habitats (B. H. McRae et al. 2008). It analyzes a circuit of parallel and serial resistors and returns, among other output, the total effective resistance. The analogy of electric current running through a circuit of resistors also applies to water-uptake by plants from the soil matrix (Van den Honert 1948, Landsberg and Fowkes (1978)). Each transition (e.g., soil-root xylem) along the water pathway is treated like an electrical (*sensu* hydraulic) resistor with a specific resistivity. Accordingly, our soil-plant system would be a circuit consisting of resistors for soil with a certain water saturation, root cortex, and root stele. In order to determine the total effective hydraulic resistance for the two root system architectures we first had to assign resistivities to each resistor (soil, cortex, and stele). Following chapters elucidate the parametrization of the resisitivities for the soil by means of the water retention curve and for the plant roots.

Water retention curve

We defined the water retention curve after (Van Genuchten 1980) which is given by:

$$\Theta = \left[\frac{1}{1 + (\alpha h)^n}\right]^{1-\frac{1}{n}} \tag{1}$$

with Θ being the soil-water saturation (ranging from 0–1), h the pressure head [cm], and α [cm^{-1}] and n soil-(texture-)specific parameters.

We set $\alpha = 0.005\ cm^{-1}$ and n = 2, which correspond to a sandy soil (Figure 1).

Given this water retention curve, we aimed to translate a given water-saturation level in a gradient of hydraulic resistivities for our soil profile of 100cm depth. This was done by taking the water potential h for a given soil-water saturation level (dotted line in Figure 2A). By adding and substracting 50 cm to h and reading out the corresponding Θ-values we obtained the gradient of soil-water saturations along our soil profile (dashed and solid red lines in Figure 2A). This was done for all water-saturation levels from 0–1 in 0.01 steps and plotted against soil depth (Figure 2B). The underlying simplification is that water is in equilibrium in the soil profile.

Hydraulic Resistivities

In a next step, the gradient of Θ is transformed into hydraulic resistivity values. Including the VanGenuchten (Van Genuchten 1980) parametrization into the Mualem model (Mualem 1976) yields:

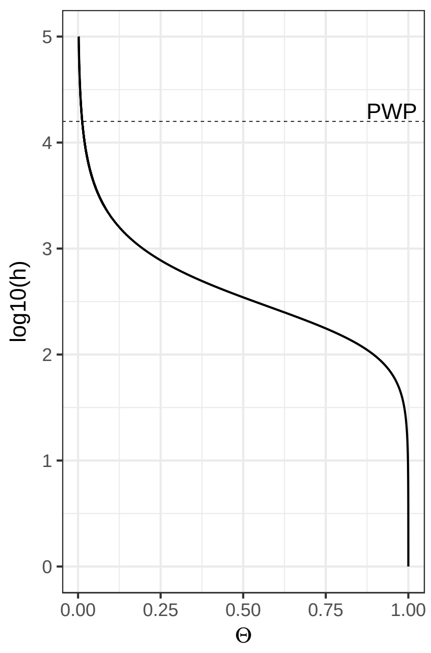

Figure 1: Water retention curve for a sandy soil used. Θ gives the relative soil-water saturation and h is the matrix potential given as the hydraulic pressure head [cm]. The dashed vertical line denotes the pressure head at the permanent wilting point (PWP) 4.2.

$$K_r(\Theta) = \Theta^{1/2}[1 - (1 - \Theta^m)^m]^2 \tag{2}$$

with $m = 1 - \frac{1}{n}$. The hydraulic resistivity of a soil raster cell is the reciprocal of the hydraulic conductivity ($R = \frac{1}{K_r}$).

The resulting resistivities ranged from 1 (at water saturation) to 43716 (water-saturation below wilting point). The resistivity gradient was scaled-up to a two-dimensional soil profile (60 x 100cm) (Figure 4).

Hydrophobicity

In this study we hypothesized that the upper 50 cm of the soil could become hydrophobic under the circumstances, that 1) a tap-rooted plant exuded mucilage into the soil and 2) mucilage dried out. Under these conditions hydrophobicity would reduce re-wetting of the topsoil. In our framework we address this mechanism by combining resistivity values of different water-saturation levels. After a re-wetting event, subsoil resistivities of depths 50–100cm are low while resistivities of the hydrophobic area at depths of 0–50cm remain high.

Root systems

The concept of our study involves water related soil-plant interactions. Therefore, we had to extend our hydraulic resistivity framework by plant-root resistivities. We chose two root system architectures to test: 1) *fibrous roots* consisting of several, surface near, adventitious roots (*fibers*), and 2) *tap roots* which are characterized by a vertical, deep growing, primary root (*tap*) and several, secondary lateral root branches.

For each root system architecture, we drew three different development-stages (Figure 4). The first stage includes the initial root development from seed germination until the first week. We assumed that both

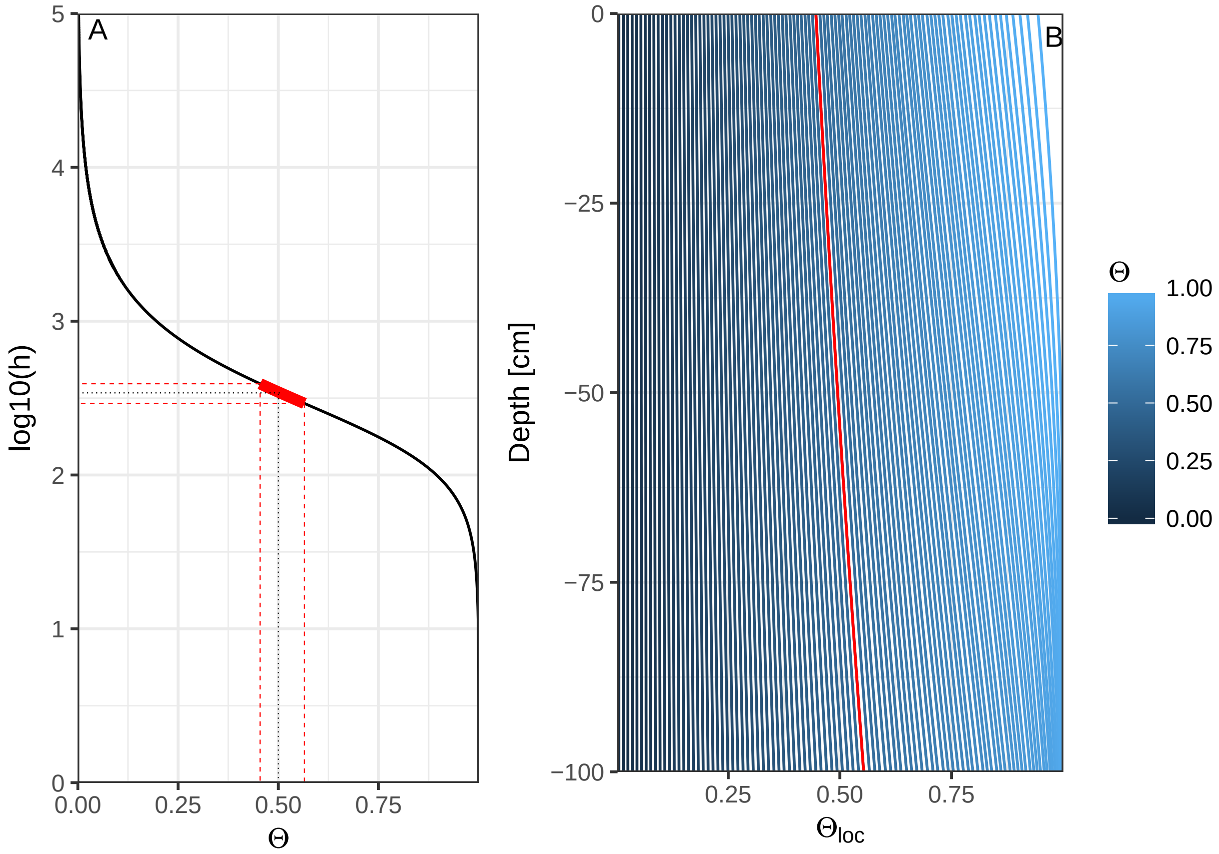

Figure 2: The assignment of resistivity values to soil matrix cells was done by the water retention curve. Here an example is given for a relative water saturation (Θ) of 0.5. The left panel (A) shows the water retention curve and the range of pressure heads being effective along a soil depth of 100 cm. The right panel (B) shows the local distribution of soil water saturation (Θ_{loc}) along the soil depth according to the effective pressure heads determined by Θ. The red line denotes the given example of $\Theta = 0.5$.

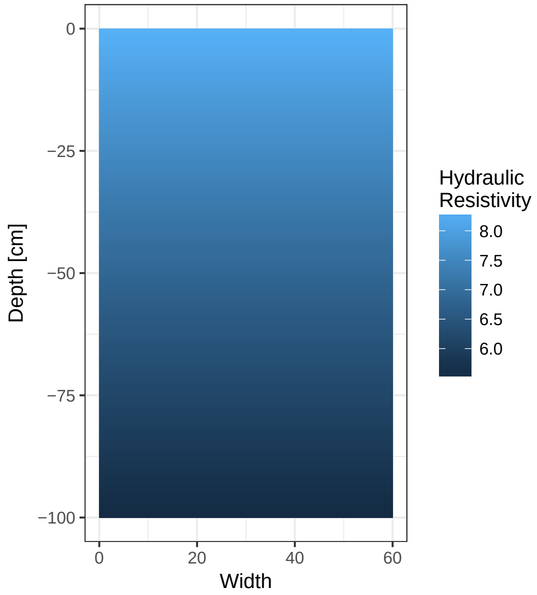

Figure 3: Vertical gradient of hydraulic resistivities along the soil profile at a water-saturation of 0.5.

architectures are similarly structured and no water uptake is necessary. The second stage is intermediate and lasts until the end of the first month. In this stage fibrous roots show all branches but not in their final length (Figure 4 A). Tap roots developed the primary vertical and the first lateral branches are developed (Figure 4 A). The last stage is the fully developed root system as shown in Figure 4 B & D.

Water flux passes several barriers along the path from below- to above-ground. Driven by water-potential gradient caused by transpiration, it needs to access the root stele via the root cortex. We used simplified sketches of the root system architectures (Figure 4) with pixel sizes of 1 mm^2. A root segment had a width of 3 pixels which were one stele pixel in the center enclosed by a cortex pixel at each side (see magnification inlet in Figure 4 C). Resisitiviy values were chosen as follows (see also Tab. 1):

1. High values for cortex cells, as water is forced to chose either an apo- or a symplastic pathway and, hence, has to cross either cell membranes or the Casparian belt. We chose values up to 1000.
2. Low values for stele cells. Here water has to be transported quickly to the above-ground shoot. Values had to be lower than the lowest soil hydraulic resisitivity of 1 (water-saturated soil) to avoid a flux of water out of the root into the soil.
3. We implemented gradients a) for cortex cells with increasing values from deep root segments to near-surface parts to address lower conductivities at old-growth roots, and b) for stele cells (again increasing from deep to surface-near) to account for water transport from lower regions in the direction of the shoot.
4. Infinite resistivity for the soil surface with the exception of one stele cell as exit for the root system to the above-ground plant parts (magnification inlet in Figure 4 C).

Table 1: Hydraulic resistivity ranges (min–max) for different types of plant raster cells.

root type	stage	stele	cortex
tap	2	$6.4e^{-3}$ – 2.5	$1.0e^{3}$ – $1.1e^{4}$
tap	3	$2.0e^{-5}$ – 1.9	$1.0e^{3}$ – $1.1e^{4}$
fib	2	$6.4e^{-3}$ – 1.3	$1.0e^{3}$ – $1.1e^{4}$
fib	3	$9.2e^{-3}$ – 1.8	$1.0e^{3}$ – $1.1e^{4}$

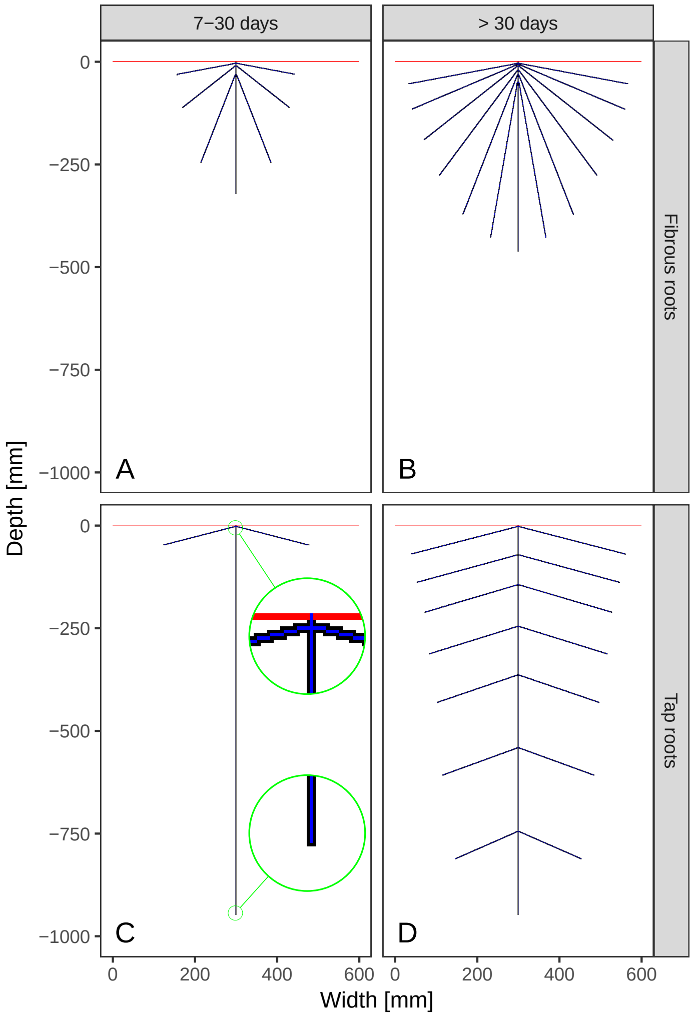

Figure 4: Root system architectures (rows) at different development stages (columns). In panel C two circular magnification inlets (green) reveal the rasterization of the systems. Roots contain cortex raster cells (black) and stele cells (blue). The surface of the system (depth = 0 mm) is sealed by a row of water-impermeable raster cells (red) with exception of one stele cell as an exit to the above-ground shoot.

Circuitscape

In a last step the whole soil-plant root system is quantified by its total effective hydraulic resistance. This was done with help of the software *Circuitscape* (V. Shah and McRae 2008). *Circuitscape* focus on quantification of landscape connectivity with manifold applications in conservation ecology. To our knowledge it has not been applied to root systems in order to test hydraulic connectivity. *Circuitscape* solves a circuit of serial and parallel resistors on a 2-dimensional surface. In our case, a resistivity value was assigned to each pixel (raster cell), either according to the local water saturation Θ_{loc} for soil pixels (see above) or according to the functional role of the plant root elements (i.e., cortex or stele). In the framework of *Circuitscape* we applied a virtual power source at the deepest soil layer (at -100cm) while the exit pixel at the transition of below- to aboveground was grounded. The remaining soil-surface was made impermeable by assigning an infinite resistivity, thus, power fluxes will aim for the grounded cell only. Along the pathway of the flux, the effective resistance is a result of the local soil water saturation Θ_{loc}, the presence of a hydrophobe zone, and the configuration of the root system.

Reference

Landsberg, J. J., and N. D. Fowkes. 1978. "Water Movement Through Plant Roots." *Annals of Botany* 42 (3): 493–508. doi:10.1093/oxfordjournals.aob.a085488.

McRae, Brad H, Brett G Dickson, Timothy H Keitt, and Viral B Shah. 2008. "Using Circuit Theory to Model Connectivity in Ecology, Evolution, and Conservation." *Ecology* 89 (10). Eco Soc America: 2712–24.

Mualem, Yechezkel. 1976. "A New Model for Predicting the Hydraulic Conductivity of Unsaturated Porous Media." *Water Resources Research* 12 (3). Wiley Online Library: 513–22.

Shah, VB, and BH McRae. 2008. "Circuitscape: A Tool for Landscape Ecology." In *Proceedings of the 7th Python in Science Conference*, 7:62–66.

Van den Honert, TH. 1948. "Water Transport in Plants as a Catenary Process." *Discussions of the Faraday Society* 3. Royal Society of Chemistry: 146–53.

Van Genuchten, M Th. 1980. "A Closed-Form Equation for Predicting the Hydraulic Conductivity of Unsaturated Soils." *Soil Science Society of America Journal* 44 (5). Soil Science Society of America: 892–98.
