# Supplementary material for: Rhizosphere hydrophobicity: A positive trait in the competition for water
Source: PLoS One. 2017 Jul 28;12(7):e0182188. doi: 10.1371/journal.pone.0182188 (PMC5533451; doi:10.1371/journal.pone.0182188)
Supplement: S2 Text — (PDF) [file pone.0182188.s002.pdf]

# S2 Text: ODD Protocol

*Thorsten Zeppenfeld*

## Preface

This document contains supporting information *S2 Text* for the publication (Zeppenfeld et al. submitted): a detailed description of the individual-based model. It follows the standard ODD (Overview, Design concepts, Details) protocol as described in (Grimm et al. 2006, Grimm et al. (2010)). All entities, variables, and constants are named according to the model code and do not follow entirely the nomenclature in the main publication text.

## Overview

### Purpose

This model serves as an investigative tool to elucidate the role of plant root traits in the competition for soil water. The ability to acquire water from the soil is a major driver in interspecific plant competition and it depends on several root functional traits. One of these traits is the excretion of gel-like compounds (mucilage) that modify physical soil properties. Mucilage secreted by roots becomes hydrophobic upon drying, impedes the rewetting of the soil close to the root, the so called rhizosphere, and reduces water availability for plants. The function of rhizosphere hydrophobicity is not easily understandable when looking at a single plant, but it may constitute a competitive advantage at the ecosystem level. We hypothesize that deep-rooted plants avoid compettiton with shallow-rooted plants by turning the top soil hydrophobic due to the release of root exudates.

To test this hypothesis we used an individual-based model to simulate water uptake and growth of two virtual plant species populations, one deep-rooted plant capable of making the soil hydrophobic and a shallow-rooted plant. We ran scenarios with different precipitation regime ranging from dry to wet (350, 700 and 1400 mm annual precipitation sum) and from high to low precipitation frequencies (1, 7, and 14 days).
Plant species abundance and biomass were chosen as indicators for competition.

### Entities, state variables, and scales

#### Entities

The model comprises plant entities which belong to two species with different root architectures: tap roots (*taproot*) and fibrous roots (*fibroot*). They are located on patches (pixels), which are considered to be homogeneous soil entities.

#### State variables

A set of attributes, i.e., variables and constants of the different entities, is given in Table 1. In the simulation system water enters by precipitation events (*precipitation_mean*) and leaves the system either by *drainage* to the groundwater or by transpiration (*transp*) of the plants. Soil water state is described by the *water-saturation*, the share of water-filled pore volume on total soil pore volume. In case of an hydrophobicity in the topsoil, soil water state is represented for topsoil (0-50cm) and subsoil (50-100cm) seperately. In this case *hpws* is the water saturation in the topsoil (**h**ydro**p**hobicity-**w**ater **s**aturation) and *water-saturation* reflects soil water condition in the subsoil.

Table 1: Overview of entity-related state variables in the model.

| | Ecological Meaning | Entity | Dimension | Typical Value Range | Used value |
|---|---|---|---|---|---|
| **Variables** | | | | | |
| water-saturation | total soil water saturation or subsoil water saturation in case of hydrophobicity | soil patch | % | 0–100 | |
| hpws | water saturation in hydrophobic topsoil (0–50cm) | soil patch | % | 0–100 | |
| drainage | soil water leaving the system | soil patch | mm | | |
| vitality | vitality at given water cotent | taproot, fibroot | % | 0–100 | |
| biomass | dry weight of plant biomass | taproot, fibroot | g | 15 | |
| transp | transpired water leaving the system | tabproot, fibroot | mm | | |
| age | plant age | taproot, fibroot | days | 1–600 | U(1,400) |
| **Constants** | | | | | |
| precipitation_mean | amount of water input per precipitation event | soil patch | mm | 0–10,000 | 1,000 |
| prec-interval | frequency of precipitation event | soil patch | days | 0–100 | 1, 7, 14 |
| maxage | life expectancy of plant | indiv. plants | days | 400–800 | N(600,100) |

All plant entitities are given a maximal life expectancy (*maxage*). Their states at every simulation step are given by an *age*, a *biomass*, and *vitality*, a value given by a species-specific function of soil water saturation. Root system architectures of both species run through developmental stages with time. In the first week (days 1–7) both species are supposed to have the same first root system architecture with one primary vertical root branch. At intermediate root development stage two (days 8–30 days) fibrous roots already show their final architecture, but branches do not penetrate deeper soil areas. Taproots in this stage establish their principal vertical root and first laterals. The final (third) root growth stage depicts the fully developed root system architecture. it is reached after one month (>30 days) of growth and lasts to the death of the individual.

#### Scales

The simulation landscape extents to 101x101 patches (~1 hectare), wherein each patch is considered to be a 1x1x1m volume of homogeneously textured soil. At temporal scale, one simulation step ('tick') comprises a time period of about a day.

## Process overview and scheduling

After initialization the model successively runs a series of soil and plant related processes (Figure 1). At the beginning of each simulation step, a precipitation event provides the system with a certain amount of water. For each soil patch a water saturation value is calculated, with respect to a potential hydrophobic regime in case a tap-rooted plant is present. According to this local soil water saturation, for every plant individual a

vitality value (*vitality*) is calculated. This value is regulatory for succeeding processes of growth, mortality and, along with others, for reproduction probability.

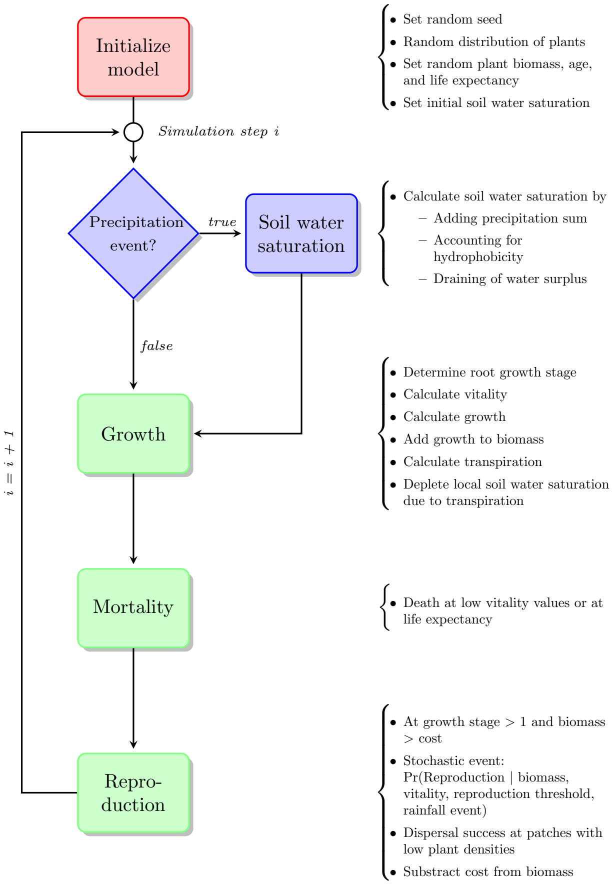


Figure 1: Basic flowchart of the model. Global process (i.e., Initialization) is colored in blue, soil related process (soil water) is filled in red and plant processes are given in green.

# Design concepts

## Basic principles

Root traits substantially determine plant's competitive performance on soil resources. In case of soil water uptake, root system architecture defines the spatial infrastructure at different depths, root lengths and complexities (i.e., branching). For the plant this is supposed to have an impact on the water uptake efficiency. Through physiological traits like the release of exudates into the rhizosphere side, the plant is able to manipulate the chemical and physical properties of the soil. One striking feature of these exudates is the hindered re-wettability of the soil after drying (Carminati et al. 2010). The ecologiocal function of this hydrophobicity in the soil is unclear.
We propose that hydrophobicity as a result of root exudate release serves as a mechanism to outcompete other plants in the competition for soil water. To test this, we implemented the model, in which taproots are able to release exudates and, hence, make topsoil water saturation less available. We implemented small-scale water-dynamics of the rhizosphere and upscale it to population level with help of an individual-based model.

### Emergence

Competitive performance of species with different traits was measured by their 1) abundance, 2) biomass, and 3) spatial distribution. It is hypothesized that soil hydrophobicity due to taproot exudates lead to lower abundances and total biomass of fibrous-rooted species and that both species separate by spatial clustering.

### Interaction

All interactions (i.e., plant-plant and plant-soil) are mediated through resources. These are soil water saturation in first line and total biomass per soil patch in second line.

### Stochasticity

Three random processes are involved in the model. First, inititalization involves random distribution of individuals, species, biomass, life expectancy are spreaded randomly over the simulation world. Second, at each simulation step a draw of a random number determines whether reproduction takes place, and third, in case of dispersal, a random angle defines the location of the seedling.

### Observation

At the end of each simulation step, total biomass and abundance per species, total soil water saturation, sum of transpiration, and sum of drained water is recorded. At time steps 500, 1000, 1500, and 3500 spatial location, species, and biomass of every individual is recorded.

## Details

Table 2: Fixed parameter set used in the model for all scenarios.

| | Ecological Meaning | Entity | Dimension | Typical Value Range | Used value |
|---|---|---|---|---|---|
| cost_hydrophobicity | assimilate costs for exudates | taproot | % of Assimilates | 0–100 | 20 |
| max_biom | maximal biomass per individuum | all plants | g | 11–1,000 | 100 |
| rep-prob | value to parameterize reproduction probability | all plants | | 1–10,000 | 1000 |
| seed_dist | dispersal radius | taproot, fibroot | m | 0–5 | 2 |
| no_seeds | number of seeds | taproot, fibroot | # | 1–10 | 1 |
| death_thrs | lowest threshold for vitality | all plants | | 0–1 | 0.2 |
| kt | water-use efficiency | all plants | $\frac{g_{H_2 0}}{g_{Biomass}}$ | 0-800 | 200 |
| porosity | ratio of pore volume to soil volume | soil | | 0–1 | 0.5 |

## Initialization

At initialization of the model each soil patch is randomly given a soil water saturation from 1–100 %. In each patch, either one taproot- or a fibroot-individual is placed (chance 50:50). Each individual is given an initital *biomass*, *age*, and life-time expectancy (*maxage*).

## Input

We parametrized a species-specific water-uptake function. It relates the plant variable *vitality* to the actual water saturation level at the location of the individual. This function is dependent on hydraulic performance of the root system architecture at different soil water saturation levels. We quantified hydraulic performance of the two different root architectures by calculating their total effective hydraulic resistance with the software *Circuitscape* (Shah and McRae 2008). This tool originates in landscape connectivity analysis. Basically, it analyses a circuit of parallel and serial resistors and returns the total effective resistance. In our case input data was a raster (600 x 1000 cells) of an idealized root system (see Fig. 2 in (Zeppenfeld et al. submitted)). In this raster each cell is assigned either to the soil, to root cortex, or to root stele. Depending on these raster cell types, the raster was filled with specific hydraulic resistivity values determined by expert knowledge (see table~3). Then, in *Circuitscape*, we calculated total hydraulic resistances for different root system architectures at different development stages at different soil water-saturations. With these total hydraulic resistances we paramterized the *vitality* function.

Table 3: Hydraulic resistivity values assigned to raster cell types.

| Type | Resistivity |
|---|---|
| dry soil (water saturation 0) | 10,000 |
| wet soil (water saturation 100) | 10 |
| cortex root cells | 57–1,000 |
| stele root cells | 1–2 |
| soil surface | $\infty$ |

Further detail on this analysis is given at Zeppenfeld et al. 2016 and its supporting information.

## Submodels

### Soil water saturation

At the beginning of each simulation step $i$ the water saturation in each soil patch is calculated. In the absence of root exudates (either deactivated or absence of taproot plants) this is straight-forward by taking the sum of the previous water saturation and the mean precipitation (*precipitation_mean*).

In case of at least one taproot plant being present in the soil patch and its trait (to release exudates and turn topsoil hydrophobic) being activated, soil-water saturation in this particular patch is calculated separately for topsoil (0–50 cm) and subsoil (50–100 cm) strata. The water input *precipitation_mean* is shared between both strata according to a distribution factor *wdist*, which is strongly affected by the amount of taproots biomass in the soil patch, assuming that taproot biomass is directly related to the amount of root releases, the volume of rhizosphere, and, hence, to the strength of the hydrophobicity-effect.

$$hpws_i = hpws_{i-1} + \frac{Precipitation_{mean}}{porosity \cdot z_{top]}} \cdot \left( \frac{1 - wdist_i}{2 - wdist_i} \right)$$

$$water - saturation_i = water - saturation_{i-1} + \frac{precipitation_{mean}}{porosity \cdot z_{sub}} \cdot \frac{1}{2 - wdist_i}$$

where $z_{top} = z_{sub} = 100$ cm is the profile depth of top- or subsoil.

The distribution factor *wdist* ranges from 0 to 1 and describes the share of the top- and subsoil on incoming precipitation water.
The more mucilage is exuded from tap-roots, the more water is repelled from this region and drained to the subsoil ($wdist \rightarrow 1$). If $wdist = 0$, precipitation water is divided half by half among top- and subsoil.

To quantify *wdist* we first related the *biomass* of taproots to the total root length $L$:

$$L = \frac{biomass}{2\pi r_{root}^2 \rho_{root}}$$

$r_{rhizo} = 0.4$ cm is the radius of rhizosphere, $r_{root} = 0.02$ cm is the radius of a root branch, and $\rho_{root} = 0.01$ $^g/_{cm^3}$ is the density of a root branch.

With total root length $L$ a rhizosphere volume can be described and *wdist* is the share of rhizosphere volume in total topsoil volume:

$$wdist = \frac{L\pi \ r_{rhizo}^2}{V_{top}}$$

Here, $r_{rhizo} = 0.4$ cm denotes the radius of the rhizosphere (soil around a root branch) and $V$_{top} = 50x100x100 cm$^3$ is the total topsoil volume. All together, we described *wdist* as follows:

$$wdist_i = \frac{\sum biomass_{taproot,i} \cdot \pi \cdot r_{rhizo}^2}{2\pi r_{root}^2 \cdot \rho_{root} \times V_{topsoil}}$$

with $\sum biomass_{taproot,i}$ being the below-ground share (0.5) of total tap-rooted plant biomass in a soil patch.

### Growth

Based on the actual water saturation in the soil patch, the *vitality* value for each individual is calculated. This is done by a species-specific water-uptake function with one argument, the local water-saturation.

In case without hydrophobicity, both *vitality* could be described with one function for plant species:

$$vitality_i = \frac{1 - e^{-k \cdot water\text{-}saturation_i}}{1 - e^{-k}} \quad (1)$$

Best fitting parameters were $k = 98.79$ for root stage two and $k = 63.88$ for root system at stage three.

When hydrophobicity affects the topsoil, a set of different parameters were found (and implemented) to describe species *vitality* at different root developmental stages best:

$$vitality_i = 1 - e^{a \cdot hpws_i + b} \cdot \frac{1}{1 + e^{c \cdot water\text{-}saturation_i + d}} \quad (2)$$

Here, four parameters had to be fitted (Tab. 4).

Table 4: Best estimates for parameters of Eq. 2.

| Root type | Stage | a | b | c | d |
|---|---|---|---|---|---|
| Fibrous | 2 | -52.70 | -0.00 | -0.0024 | -3.49 |

| Root type | Stage | a | b | c | d |
|---|---|---|---|---|---|
| Tap | 2 | -33.17 | -2.35 | -118.71 | 0.32 |
| Fibrous | 3 | -48.64 | -0.29 | -30.08 | -0.74 |
| Tap | 3 | -20.00 | -2.96 | -15.16 | -3.03 |

Growth of each individual is based on its *vitality* value, its *biomass* and the maximal biomass *max_biom*:

$$biomass_i = biomass_{i-1} + \frac{vitality_i}{50} \times \left(1 - \frac{biomass_{i-1}}{max\_biom}\right) \quad (3)$$

The growth of *biomass* is accompanied by loss of water due to transpiration. Here the coefficient of transpiration *kt* is multiplied by biomass increment and a basic transpiration rate of 0.2 per unit *biomass* is added. Transpiration water is substracted from soil water saturation. In case of hydrophobicity in the topsoil, transpiration water sum per patch is substracted equally from topsoil and subsoil.

### Mortality

At each simulation step *vitality* and *age* of all individuals are checked. If *vitality* is below the mortality threshold *death_ths* and/or *age* exceeds life expectancy *maxage* the individual dies.

### Reproduction & Dispersal

Each individual which passed 60 ticks (~two months) is assumed to be adolescent and could potentially reproduce. The chance of reproduction—hence dispersal—is checked by drawing a random number from 0 to *rep_threshold*. If this number is less than the product of *biomass* and *vitality*, a dispersal event is commenced. So well-grown individuals and/or vital plants have a higher chance to reproduce. Dispersal is simulated by a simplified gaussian kernel, where a random angle was chosen and the dispersal distance is a realization of a Gaussian normal distribution with $\mu = 0$ and $\sigma = seed\_dist$. The success of a dispersal event is dependent on plant density and driven by the total biomass of the target patch. If the totla biomass does not exceed 50g, a new seedling is initialized with $biomass = 1$. This amount of *biomass* is invested by the reproducing individual and is substracted from its *biomass*.

## Annotation

This document was created by using RMarkdown (Allaire et al. 2016) in RStudio (RStudio Team 2012).

## References

Allaire, JJ, Joe Cheng, Yihui Xie, Jonathan McPherson, Winston Chang, Jeff Allen, Hadley Wickham, Aron Atkins, and Rob Hyndman. 2016. *Rmarkdown: Dynamic Documents for R*. https://CRAN.R-project.org/package=rmarkdown.

Carminati, Andrea, Ahmad B. Moradi, Doris Vetterlein, Peter Vontobel, Eberhard Lehmann, Ulrich Weller, Hans-Jörg Vogel, and Sascha E. Oswald. 2010. "Dynamics of Soil Water Content in the Rhizosphere." *Plant and Soil* 332 (1-2). Springer Netherlands: 163–76. doi:10.1007/s11104-010-0283-8.

Grimm, Volker, Uta Berger, Finn Bastiansen, Sigrunn Eliassen, Vincent Ginot, Jarl Giske, John Goss-Custard, et al. 2006. "A Standard Protocol for Describing Individual-Based and Agent-Based Models." *Ecological*

*Modelling* 198 (1-2): 115–26. doi:http://dx.doi.org/10.1016/j.ecolmodel.2006.04.023.

Grimm, Volker, Uta Berger, Donald L. DeAngelis, J. Gary Polhill, Jarl Giske, and Steven F. Railsback. 2010. “The ODD Protocol: A Review and First Update.” *Ecological Modelling* 221 (23): 2760–8. doi:http://dx.doi.org/10.1016/j.ecolmodel.2010.08.019.

RStudio Team. 2012. *RStudio: Integrated Development Environment for R*. Boston, MA: RStudio, Inc. http://www.rstudio.com/.

Shah, VB, and BH McRae. 2008. “Circuitscape: A Tool for Landscape Ecology.” In *Proceedings of the 7th Python in Science Conference*, 7:62–66.

Zeppenfeld, Thorsten, Niko Balkenhol, Kristof Kovacs, and Andrea Carminati. submitted. “Rhizosphere Hydrophobicity: A Positive Trait in the Competition for Water.” *PLOS ONE*.
