# Supplementary figures and images for: Rhizosphere hydrophobicity: A positive trait in the competition for water
Source: PLoS One. 2017 Jul 28;12(7):e0182188. doi: 10.1371/journal.pone.0182188 (PMC5533451; doi:10.1371/journal.pone.0182188)

A: Total precipitation sum: 350 mm

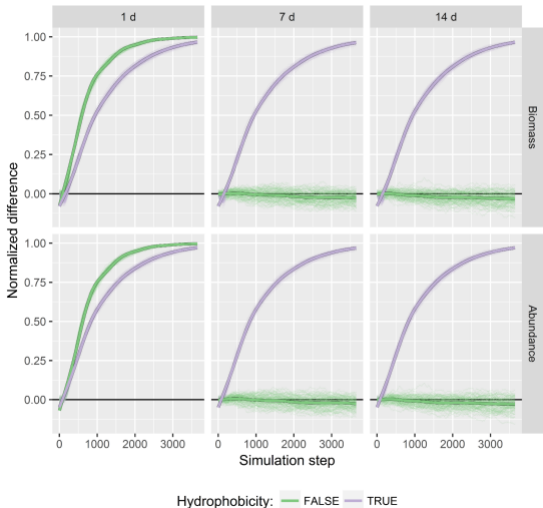

B: Total precipitation sum: 700 mm

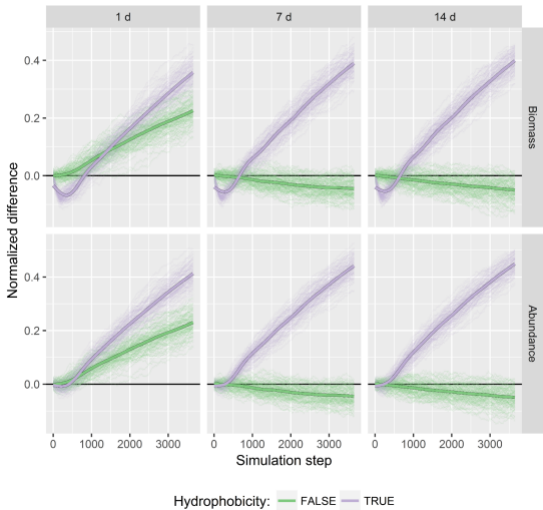

C: Total precipitation sum: 1400 mm

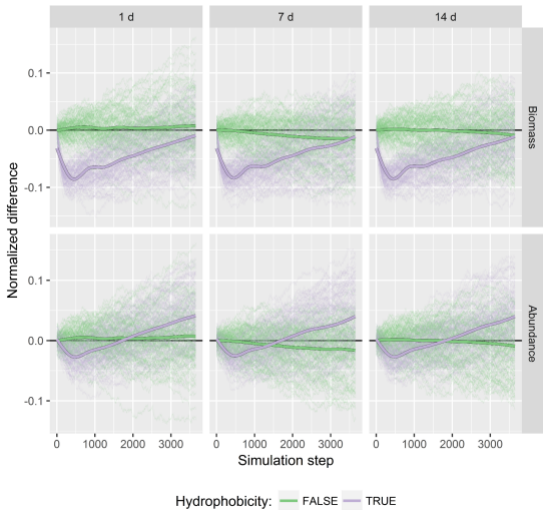

Supplement: S1 Fig — Columns show different precipitation frequencies. Rows show population indices total biomass (top panel) and total abundance (bottom panel). Precipitation sum is 350 mm (A), 700 mm (B), and 1400 mm (C). Values are differences between tap-rooted to fibrous rooted plants. Differences are normalized by the total sum. Positive values are in favor of tap-rooted plants. Thin lines are results of 100 simulation runs with either hydrophobicity trait activated (violet) or deactivated (green). Thick lines are smoothed (spline) averages. (PDF) [file pone.0182188.s004.pdf]
